# Antibacterial and Antioxidant Compounds from the Root Extract of *Aloe debrana*

**DOI:** 10.1155/2024/6651648

**Published:** 2024-05-07

**Authors:** Tokuma Getahun, Joydeep Das, Parames C. Sil, Neeraj Gupta

**Affiliations:** ^1^Department of Chemistry, Asella College of Teachers Education, Asella, Oromia, Ethiopia; ^2^Department of Chemistry, Physical Sciences, Mizoram University, Aizawl, Mizoram 796004, India; ^3^Division of Molecular Medicine, Bose Institute, P-1/12, CIT Scheme VII M, Kolkata 700054, India; ^4^Department of Chemistry and Chemical Sciences, Central University of Himachal Pradesh, Dharamshala, Kangra 176215, HP, India

## Abstract

This study was conducted to isolate and identify the chemical compounds from the roots of *Aloe debrana* (L.) and evaluate their antioxidant and antibacterial activities. From the acetone (99.5%) extract of the roots of this plant, four anthraquinones, such as chrysophanol (**1**), asphodeline (**2**), aloesaponarin I (**5**), and laccaic acid D-methyl ester (**6**), and a new catechol derivative, 5-allyl-3-methoxybenzene-1,2-diol (**3**), were isolated and elucidated by different chromatographic and spectroscopic methods together with linoleic acid (**4**), respectively. Compounds **2**, **3,** and **4** were reported here for the first time from this plant and compound **3** from the genus *Aloe*. The compounds were evaluated for their antioxidant activity using H_2_O_2_ and DPPH assays and bactericidal activity against *S. aureus* and *E. coli*. Compounds **3** and **6** showed highest antioxidant activities with IC_50_ values of 19.38 ± 0.64 and 32.81 ± 0.78 *μ*g/mL in DPPH, and 28.52 ± 1.08 and 27.31 ± 1.46 *μ*g/mL in H_2_O_2_, respectively. The isolated compounds also demonstrated considerable activity towards *S. aureus*. Among these compounds, compound **3** exhibited the highest activity (91.20 ± 0.12% and 9.14 ± 0.93 mm at 1.0 mg/mL) against this bacterium. The overall results suggest that the isolated compounds may be considered as potential sources of the bioactive agents to be used in the pharmacological, food, and other industries. Moreover, their high sensitivity against *S. aureus* may also support the use of *A. debrana* plant in the traditional medicine to treat wounds. Therefore, the isolated compounds are responsible for medicinal properties of this plant.

## 1. Introduction

*Aloe* (Family: Asphodelaceae) has found wide recognition for its medicinal and cosmetic uses [1]. Many researchers from different countries have shown interest to study on *Aloe* species because of their bioactive compounds [2], which are responsible for medicinal properties of the plants and many-sided activities. The genus is widespread in the Madagascar, Arabian Peninsula, Jordan, various Indian Ocean islands, and many African countries, and its few species are cultivated in Japan, India, Australia, America, Hawaiian Islands, Caribbean, and Mediterranean regions [3–5]. Approximately 83 *Aloe* plants occur in Eastern Africa [6], of which 46 grow naturally in dry and grasslands of Ethiopia with 16 of them being endemic [7]. *Aloe debrana* Christian is a stemless evergreen endemic medicinal *Aloe* plant of Ethiopia, which commonly grows in the areas of grassland on thin soil overlying basalt, usually on gentle slopes between 2,400 and 2,700 m above sea level in Shewa, Gojam, and Wello regions [4, 8].

In Ethiopian traditional herbal medicines, *A. debrana* is used for the treatment of wounds, eye inflammation, malaria, excessive pain, gastrointestinal, and dermatological problems [8]. It is useful in water and soil conservation [9], to stop breastfeeding, and it was examined as good thickening agent for printing polyester and cotton with disperse dyes. The leaf latex of *A. debrana* is used traditionally as laxative, antidiabetic, and antimalarial agents. It is also used for cleansing the blood, healing of wounds, and cleaning of eyes injured accidently [4]. Farmers also use this to cure the wound of the nape of their oxen made during plough [7].

Previously, various types of natural compounds, such as alkaloids, anthraquinones, pre-anthraquinones, naphthoquinones, anthrones, oxanthrones, steroids, chromones, pyrenes, and flavonoids, were isolated from *Aloe* plants [2, 5]. Moreover, only few compounds were identified from *A. debrana* plant. Therefore, the objectives of this work were to isolate compounds from the acetone extract of *A. debrana* roots and elucidate their structure by using chromatographic and spectroscopic methods, respectively. In addition, the antioxidant and antibacterial potentials of the compounds, which may be useful in foods, pharmaceuticals, and other industries, were also assessed and reported.

## 2. Materials and Methods

### 2.1. General

Column chromatography (CC): silica gel 200–400 mesh Merck. Sephadex chromatography (SC): LH-20 (200 g). Thin-layer chromatography (TLC): a ready-made 0.2-mm-thick layer of silica gel GF_254_ (Merck) coated on aluminium plate: detection by UV light at 254 nm, and by using vanillin solution and heating for few minutes or by using iodine vapour. UV-Vis spectra were recorded on Perkin-Elmer Lambda 750 UV/VIS NIR spectrophotometer (200–600 nm). IR spectra were obtained by Perkin-Elmer Spectrum 400 FT-IR/FT-FIR spectrometer. NMR spectra were performed on a Bruker Avance Neo 500 MHz NMR spectrometer in either CDCl_3_ or DMSO-d_6_ solutions with TMS as internal standard.

### 2.2. Plant Material

The fresh roots of *A. debrana* were collected from Kube Bedesa Koricho, Weliso Woreda, Oromia, Ethiopia, which is 117 km far from south-west of Addis Ababa near Weliso town (located at 8°32′N 37°58′E latitude and longitude, respectively) in April 2019. The plant material was authenticated by Professor Legesse Negash, and a voucher specimen (No. 00A1) was deposited at Ethiopian National Herbarium of the Addis Ababa University.

### 2.3. Extraction and Isolation

The powdered *A. debrana* Christian roots (300 g) were extracted with 99.5% acetone (1.5 L) using maceration for 3 days at room temperature (22 ℃) as described in the paper published by Melaku et al. [10]. It was filtered and concentrated to afford 3.81 g (1.27%) reddish brown jelly crude residue. This crude extract (3.27 g) was subjected to the CC on silica gel (200–400 mesh, 180 g) using gradient flow of EtOAc in *n*-hexane (100 : 0 ⟶ 0 : 100) to yield 21 fractions (Fr. 1–Fr. 21) each 100 mL, which were combined based on their TLC profile as Fr. 1–5, Fr. 6–7, Fr. 8–9, Fr. 10–13, Fr. 14–17, and Fr. 18–21. Fr. 1–5 (546 mg) was re-chromatographed over silica gel (25 g) using gradient solvent system of *n*-hexane/EtOAc (100 : 0 ⟶ 0 : 60) to furnish eight fractions (Fr. 1.1–Fr. 1.8) each 10 mL. Fr. 1.4 to 1.6 were combined (193 mg) and subjected to silica gel (15 g) CC (*n*-hexane/EtOAc) to obtain compound **1** (13.25 mg). Fr.8-9 (385 mg) was applied to silica gel (20 g) CC eluted with *n*-hexane/EtOAc (100 : 0 ⟶ 0 : 50) to yield five fractions (Fr.2.1–Fr.2.5) each 20 mL. Fr. 2.2 (146 mg) was passed through silica gel (15 g) CC (*n*-hexane/EtOAc, 100 : 0 ⟶ 0 : 60), followed by Sephadex LH-20 (CH_2_Cl_2_/MeOH, 1 : 1 v/v) to get compound **2** (9.18 mg) and compound **3** (8.32 mg). Similarly, Fr. 2.5 (92 mg) was re-chromatographed over 15 g of silica gel using increasing gradient of *n*-hexane/EtOAc to give compound **4** (15.06 mg). Fr. 10–13 (869 mg) was separated using silica gel (30 g) CC, eluted with *n*-hexane/EtOAc (100 : 0 ⟶ 0 : 80), to afford 12 fractions (Fr. 3.1–Fr. 3.12) each 10 mL. Fractions 3.4 to 3.8 were combined (371 mg) and subjected to repeated silica gel CC (*n*-hexane/EtOAc) to get compound **5** (31.67 mg). Finally, Fr. 14–17 (611 mg) was applied to silica gel (25 g) CC eluted with *n*-hexane/EtOAc (100 : 0 ⟶ 0 : 100), to yield compound **6** (53.82 mg).

### 2.4. Antioxidant Activities

Antioxidant activities of the isolated compounds were evaluated by using DPPH and H_2_O_2_ assays at the final concentrations within the range of 31.25 to 1000 *μ*g/mL. Ascorbic acid, a well-known antioxidant compound, was used as a positive control in all the assays. DPPH and H_2_O_2_ were obtained from School of Pharmacy of the Faculty of Pharmaceutical Sciences, Shoolini University, India. The assay was also carried out at this school.

#### 2.4.1. DPPH Assay

The antioxidant properties of the isolated compounds were determined by DPPH assay [11]. Three millilitres of standard solution of each of the concentrations from 31.25 to 1000 *μ*g/mL was mixed with 1.0 mL of 90 *μ*M DPPH solution in MeOH to make the test solutions. Ascorbic acid was prepared in same way as the test samples. A mixture of 3 mL of MeOH and 1 mL of DPPH solution was used as negative control. Each assay was performed three times, and the prepared samples were incubated in the dark at 37℃ for about 30 min; then, the absorbance for each was determined at a wavelength of 515 nm using a spectrophotometer. Antioxidant activity of all the test samples was expressed as IC_50_ (*μ*g/mL).

#### 2.4.2. H_2_O_2_ Assay

The scavenging activity of the isolated compounds was also investigated three times by H_2_O_2_ assay [12]. The concentrations from 31.25 to 1000 *μ*g/mL of each of the test samples and the reference antioxidant compound, and ascorbic acid in deionized water was dissolved in 3.4 mL of 0.10 M phosphate buffer of pH 7.4 and mixed with 0.60 mL of 40 mM H_2_O_2_ solution. After few minutes, the absorbance of the mixture was determined at 230 nm using a spectrophotometer. Negative control was prepared by replacing the test samples with distilled water. Antioxidant activity of all test samples was expressed as IC_50_ (*μ*g/mL).

### 2.5. Bacterial Growth Inhibition Assay

The bacterial growth inhibition assay of the isolated compounds was performed using cultures of the Gram (+) (*Staphylococcus aureus* ATCC 25923) and the Gram (−) (*Escherichia coli* ATCC 25922). These strains were obtained from KPC Medical College, and the assay was carried out at the Bose Institute, Kolkata, India. Weighed aliquots of each dry sample were dissolved in DMSO to give different concentrations (0.25, 0.50, 0.75, and 1.0 mg/mL). From an overnight grown culture in Luria–Bertani (LB) broth media at 37℃, each of 5, 10, 15, and 20 *μ*L of inoculums was separately added to 1 mL fresh culture medium. LB with only samples was considered as blank and LB with only inoculums as controls in the experiments. All the test samples were then incubated for about 48 h at 37℃. Finally, the growth of the bacteria was measured using a UV-Vis spectrophotometer at 600 nm [13]. The sensitivity of the bacterial species to the samples was determined by measuring the percent inhibition of the bacterial growth.

Additionally, disc diffusion analysis was also performed according to the National Committee for Clinical Laboratory Standards (NCCLS) [14] against the same pathogens, to assess the bactericidal activity of the compounds **3** and **6**, which showed good antibacterial activity using the method previously described. For this method, 6-mm-diameter sterilized Whatman No. 1 filter paper discs were saturated with different concentrations (0.50 and 1.0 mg/mL) of these samples and placed on nutrient agar (NA) plates. The plates were pre-inoculated with each of the test strain in suspension (10^7^-10^8^ CFU/mL) of bacteria and then incubated for about 24 h at 37 ℃. After incubation, diameters of their inhibition zones (DIZ) in millimetres were measured. The antibiotic gentamicin was used as a control (positive) against the selected bacterial strains.

### 2.6. Statistical Analysis

All experimental results were expressed as mean value and standard deviation (*x* ± SD) of repeated trials (three times for all) and determined using Excel software. The IC_50_ values were also determined using Excel software by plotting inhibition-concentration curves. A comparison of the group means and the difference between the groups (*p* values <0.05) were verified by Student's *t*-test.

## 3. Results and Discussion

### 3.1. Characterization of the Isolated Compounds

Structure elucidation of the compounds was performed by employing various spectroscopic techniques and by comparing with spectral data reported for the same compounds. Compound **1** was isolated as yellow amorphous solid. By comparing its physical properties, UV (MeOH), IR (KBr, *v*), and NMR data with the literature values, the compound was identified as chrysophanol [15]. Chrysophanol is a known anthraquinone (phenolic compound) isolated from various organs and species, which shows diverse biological activities that include antimutagenic, anti-inflammatory, antiprotozoal, immuno-stimulatory, spasmolytic, antidiabetic, antigenotoxic, and antimicrobial effects [6, 10, 16, 17]. It is also active against HIV-1 protease and inhibits the replication of poliovirus, induced necrosis in human liver cancer cells, and well-known potent photosensitizer [10, 17]. Compound **2** was obtained as orange powder. By comparing its physical properties, UV (MeOH), IR (KBr, *v*), and NMR data with the literature values, the compound was identified as asphodeline [18].

**Figure 1:**
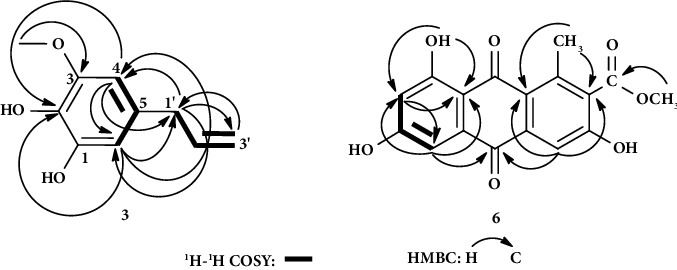
^1^H-^1^H COSY and strong HMBC correlations of compounds **3** and **6**.

**Figure 2:**
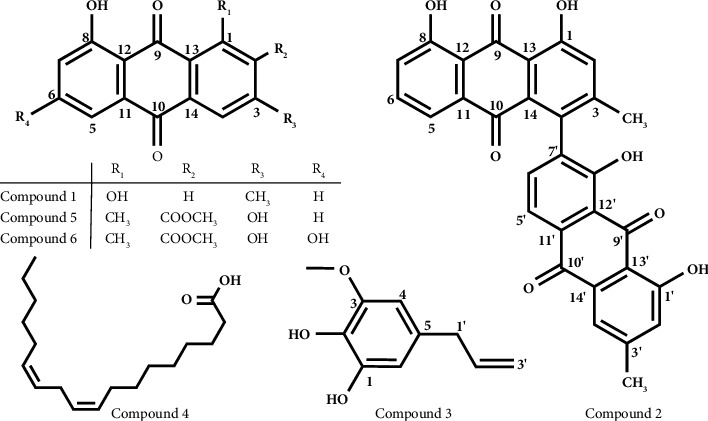
Structures of compounds isolated from *A. debrana* roots.

Compound **3** was isolated as pale yellow jelly substance with molecular formula of C_l0_H_l2_0_3_ by HR-MS ([M]^+^ = m/z 180.1) analysis. Its UV spectrum (CHCl_3_) exhibited absorption maxima at 236 and 240 nm. Its IR (KBr, *v*) spectrum showed the presence of hydroxyl group (3512 cm^−1^), aromatic ring (1638, 1438 cm^−1^), and alkene (1606 cm^−1^) functionalities. Its ^13^C-NMR and DEPT-135 spectra displayed four sp^2^ quaternary carbons, three sp^2^ methines, one sp^3^ methylene, one sp^2^ methylene, and one oxygenated methyl. ^1^H-NMR spectrum (500 MHz, CDCl_3_) displayed the presence of two *meta*-coupled aromatic nonequivalent methine protons with small coupling constant at *δ*_H_ 6.30 (1H, *d*, *J* = 1.5 Hz, H-4) and 6.44 (1H, *d*, *J* = 2.0 Hz, H-6), one oxygenated methyl protons at *δ*_H_ 3.86 (3H, *s*), and olefinic methine proton at 5.94 (1H, m, H-2′) (Table 1). Two doublet signals at *δ*_H_ 3.28 (2H, *d*, *J* = 7 Hz) and 5.06 (1H, *d*, *J* = 1.5 Hz) assigned to H-1′ and H-3′ a, respectively. HMBC correlation from -OCH_3_ protons to C-3 confirmed the position of the methoxy group. Another strong correlations observed were between methylene (-CH_2_-) protons at *δ*_H_ 3.28 (H-1′) with the carbons at *δ* 103.4 (C-4), 108.8 (C-6), and 115.7 (C-3′) establishing the site of attachment of the allylic group to C-5 of benzene ring (Figure 1). The COSY spectrum also showed correlations between meta-coupled aromatic protons, H-4 (*δ* 6.30) and H-6 (*δ* 6.44), and between allylic protons (Figure 1). Thus, compound **3** was elucidated as catechol derivative, 5-allyl-3-methoxybenzene-1,2-diol (Figure 2). To the best of our knowledge, this compound is not isolated from plants. However, as reported by earlier researchers, it was synthesized as a major oxidative product of myristicin [19]. Catechol was isolated from the dichloromethane extract of *A. ferox* [20].

Compound **4** was isolated as colourless oil. By comparing the physical properties, IR and NMR data of this compound with the literature values, it was identified as linoleic acid [21]. Linoleic acid is a known useful unsaturated (omega-6) fatty acid that has been reported from various medicinal plants, including *Artemisia integrifolia* L. [22] and *Mesua ferrea* L. [23]. Compound **5** was obtained as yellow powder. By comparing physical properties, UV (MeOH), IR (KBr, *v*), and NMR data of this compound with those reported in the literature, it was identified as aloesaponarin I and it is reported to show moderate antiplasmodial activity [6, 24].

Compound **6** appeared as a yellow amorphous solid. Its UV spectrum (MeOH) exhibited absorption maxima at 219, 285, 345, and 433 nm, the typical characteristic of anthraquinones [24]. Its IR (KBr, *v*) spectrum showed the presence of hydroxyl group (3404 cm^−1^), aromatic ring (1568, 1441 cm^−1^), ester carbonyl (1728 cm^−1^), and ketone carbonyl (1639 cm^−1^) functionalities. ^1^H-NMR spectrum (500 MHz) of this compound (Table 2) showed one chelated OH group at *δ*_H_ 13.10, two methyls, and only three aromatic nonequivalent methine protons. These methine protons are *meta*-coupled protons at *δ*_H_ 7.07 (1H, *d*, *J* = 2.5 Hz, H-5) and *δ*_H_ 6.60 (1H, *d*, *J* = 2.5 Hz, H-7), consistent with the presence of OH group at C-6, and the third proton at *δ*_H_ 7.60 (1H, *s*, H-4) was assigned to H-4 of a 1,2,3-tri-substituted benzene ring. Evidence of a substituent at C-1 was deduced from the presence of a methyl (*δ*_H/C_ 2.61 (*s*, 3H)/20.3). ^13^C-NMR spectrum (Table 2) along with DEPT-135 displayed 17 carbon signals as in compound **5**. In the same way as compound **5**, the presence of methyl ester at C-2 was confirmed from a methoxy signal at *δ*_H/C_ 3.87 (*s*, 3H)/53.0 and *δ*_C_ 167.7 for ester carbonyl. The only difference of the two is that, in compound **6**, one of the aromatic ring methines of compound **5** was changed to oxygen-bearing aromatic methine carbon (*δ*_C_ 164.7, C-6). This and the connectivity of the protons and carbon resonances of **6** were also supported by a series of the 2D-NMR (^1^H-^1^H COSY, HSQC, and HMBC) spectra. There is a correlation only between H-5 (*δ* 7.07) and H-7 (*δ* 6.60) in the COSY spectrum, indicating the absence of a proton on C-6 and H-4 is on the tri-substituted anthraquinone benzene ring (Table 2, Figures 1 and 2). The HMBC spectrum of this compound showed strong correlation between chelated OH group proton at *δ* 13.10 with the carbons at *δ* 108.8 (C-7), 165.0 (C-8), and 110.7 (C-12) establishing the site of attachment of the chelated OH to C-8 of benzene ring. Another key strong correlation observed was between aromatic proton signal of tri-substituted anthraquinone benzene ring at *δ* 7.60 (H-4) with methyl ester substituted aromatic carbon signal at *δ*_C_ 130.2 (C-2), ketone carbonyl carbon at *δ* 182.4 (C-10), and other aromatic carbon signal at *δ* 123.0 (C-13), which established the site of attachment of methyl ester to the aromatic ring (Figure 1). The NMR spectral data of compound **6** were found in agreement with the NMR spectral data reported in the literature for laccaic acid D-methyl ester [25]. Laccaic acid D-methyl ester is an anthraquinone (phenolic compound) previously identified from *A*. *secundiflora* roots and reported that it has no cytotoxicity [6]. Compounds **2**, **3,** and **4** were reported here for the first time from this plant and **3** from the genus *Aloe* and other plants.

### 3.2. Antioxidant Activities of the Isolated Compounds

The antioxidant activities of the isolated compounds are presented in Table 3. Compounds **3** and **6** showed highest antioxidant activities with IC_50_ values of 19.38 ± 0.64 and 32.81 ± 0.78 *μ*g/mL in DPPH, and 28.52 ± 1.08 and 27.31 ± 1.46 *μ*g/mL in H_2_O_2_, respectively. Compound **5** also exhibited relatively high antioxidant activity with IC_50_ values of 57.24 ± 1.07 *μ*g/mL in DPPH, and 49.34 ± 1.33 *μ*g/mL in H_2_O_2_. However, compounds **1** and **2** exhibited lowest activities. The high antioxidant activity of compound **3** may be due to its hydroxyl groups, and that of compounds **5** and **6** may be attributed to their number of hydroxyl and carbonyl groups as clearly discussed in the study published by Ben Ammar et al. [26].

According to the available literature, phenolic compounds or their derivatives were reported to exhibit antioxidant activities [27]. Researchers demonstrated that chrysophanol has no activity against DPPH and ABTS^+^ radicals [28]. However, other researchers showed that the compound had a scavenging effect on DPPH radical (IC_50_ value of 26.56 *μ*g/mL). This big difference may be from the errors in the operation and the excessive differences in experimental conditions [29]. When compared to the results obtained in our study, compound **1** and its dimer (**2**) were less active against the radicals. However, to the best of our knowledge and according to literature survey, there was no previous antioxidant activity report for other compounds.

### 3.3. Bacterial Growth Inhibition of the Isolated Compounds

In Tables 4 and 5, results of the bactericidal activities (bacterial growth inhibition) of the isolated compounds against the investigated strains of bacteria are shown. Percent inhibition of the bacterial growth demonstrated that all the compounds inhibited the mean growth of a Gram (+) bacterium, *S. aureus* (13.82 ± 0.27 to 91.20 ± 0.12% inhibition), whereas they showed weak growth inhibition of *E. coli* (3.06 ± 1.10 to 7.18 ± 1.01% inhibition) evaluated at the final concentrations within the range of 0.25 to 1.0 mg/mL. Among the identified compounds, the highest inhibition was observed for compounds **3** and **5** against the growth of *S. aureus* in all the tested concentrations. The diameter of inhibition zones (DIZ) of compounds **3** and **5** was 6.87 ± 0.93 and 9.14 ± 0.93 mm, as well as 6.55 ± 0.87 and 8.21 ± 1.24 mm for *S. aureus* at 0.5 and 1.0 mg/mL concentrations, respectively. The results indicate the susceptibility of this bacterium to the compounds. Moreover, compound **3** demonstrated activity (2.91 ± 1.06 mm) towards *E. coli* at 1.0 mg/mL. However, compound **5** showed no activity towards *E. coli* at both concentrations.

Phenolic compounds or their derivatives were reported to show antibacterial activities [27]. Literature searches on the antibacterial activities of the isolated compounds in the present study indicated that compounds **1** and **5** have been reported to possess antibacterial activities [10, 30]. The result for compound **1** is almost comparable (moderately active) with the results reported for the same compound against *S. aureus* with DIZ of 10 mm at 50 mg/mL [30] and 13 mm at 1.0 mg/mL [10]. It was also moderately active against *B*. *subtilis* (DIZ, 10 mm), *K. pneumoniae* (DIZ, 11 mm), and *P*. *aeruginosa* (DIZ, 12 mm) [10, 30], but showed no activity towards *E. coli* [30] and *P. mirabilis* [10]. On the other hand, Abdissa et al. [30] reported the greatest antibacterial potential for compound **5** evaluated at 50 mg/mL concentration. The compound was highly active towards *B. subtilis* (DIZ, 27 mm) than the reference antibiotic drug, gentamicin (DIZ, 25 mm). It was also more active against *E. coli*, *P*. *aeruginosa*, and *S. aureus* with DIZ of 22, 21, and 18 mm, respectively. When compared to the results obtained in our study, this compound was less active (8.21 ± 1.24 mm) against *S. aureus* and not active towards *E. coli* at 1.0 mg/mL. These variations may be due to the concentrations used for testing the activities. However, to the best of our knowledge, there is no prior report on antibacterial activity of compounds **2** and **3** against any bacterial strains and compound **6** against *S. aureus* and *E. coli*.

## 4. Conclusion

In this study, six compounds were isolated and elucidated from *A. debrana* roots. Compounds **2**, **3,** and **4** were reported here for the first time from this plant and compound **3** from the genus *Aloe* and other plants. The compounds such as **3**, **5,** and **6** exhibited high antioxidant activities. In addition, the tested compounds demonstrated appreciable growth inhibition of *S. aureus*. Among them, the highest inhibition observed was for compounds **3** and **5**. However, no significant activity was reported for any of the isolated compounds against *E. coli.* The overall results suggest that the isolated compounds may be useful in foods, pharmaceuticals, and other industries. Moreover, their high sensitivity against *S. aureus* may also support the use of *A. debrana* plant in the traditional medicine to treat wounds. Therefore, the isolated compounds are responsible for medicinal properties of this plant. Furthermore, studies on *in vivo* efficacy tests and toxicity of *A. debrana* plant would be required to ensure its use for the treatment of wounds and other different ailments (supplementary files) (available here).

## Figures and Tables

**Table 1:** ^1^H (500 MHz, CDCl_3_), ^13^C (125 MHz, CDCl_3_), and DEPT-135 (125 MHz, CDCl_3_) spectral data for compound **3** isolated from *A*. *debrana* roots.

Position	^1^H-NMR (*δ* ppm, *m*, *J* in Hz)	^13^C-NMR (*δ* ppm)	DEPT^a^-135
1	—	143.8	C
2	—	130.6	C
3	—	146.8	C
4	6.30 (*d*, *J* = 1.5 Hz, 1H)	103.4	CH
5	—	132.0	C
6	6.44 (*d*, *J* = 2.0 Hz, 1H)	108.8	CH
1′	3.28 (*d*, *J* = 7 Hz, 2H)	40.1	CH_2_
2′	5.94 (m, 1H)	137.5	CH
3′a	5.06 (*d*, *J* = 1.5 Hz, 1H)	115.7	CH_2_
3′b	5.10 (*d*, *J* = 1.5 Hz, 1H)		
OCH_3_	3.86 (*s*, 3H)	56.1	CH_3_

^a^DEPT: Distortionless Enhancement by Polarization Transfer.

**Table 2:** ^1^H (500 MHz) and ^13^C (125 MHz) NMR spectral data for compounds **1**, **5,** and **6** isolated from *A*. *debrana* roots.

Position	1 (CDCl_3_)		5 (DMSO-d_6_)		6 (DMSO-d_6_)	
	*δ*_H_ (*m*, *J* in Hz)	*δ*_*C*_	*δ*_H_ (*m*, *J* in Hz)	*δ*_*C*_	*δ*_H_ (*m*, *J* in Hz)	*δ*_*C*_
1		162.5		141.5		141.1
2	7.11 (br. *s*, 1H)	124.6		130.1		130.2
3		149.4		159.3		158.8
4	7.67 (br. *s*, 1H)	120.0	7.62 (*s*, 1H)	112.5	7.60 (*s*, 1H)	112.6
5	7.82 (*dd*, *J* = 1.0, 7.5 Hz, 1H)	137.0	7.65 (*dd*, *J* = 7.5, 1.5 Hz, 1H)	118.9	7.07 (*d*, *J* = 2.5 Hz, 1H)	107.7
6	7.67 (*m*, 1H)	124.4	7.74 (*t*, *J* = 8 Hz, 1H)	136.6		164.7
7	7.31 (*dd*, *J* = 1.5, 8.5 Hz, 1H)	121.4	7.35 (*dd*, *J* = 8.5, 1.5 Hz, 1H)	125.0	6.60 (*d*, *J* = 2.5 Hz, 1H)	108.8
8		162.8		161.9		165.0
9		192.6		189.8		188.3
10		182.1		182.3		182.4
11		133.7		132.9		137.1
12		115.9		117.3		110.7
13		113.7		123.1		123.0
14		133.3		137.3		134.6
CH_3_	2.47 (*s*, 3H)	22.3	2.62 (*s*, 3H)	20.3	2.61 (*s*, 3H)	20.3
OCH_3_			3.88 (*s*, 3H)	53.0	3.88 (*s*, 3H)	53.0
CO				167.6		167.7
1-OH	12.14					
8-OH	12.03		12.80		13.10	

**Table 3:** Antioxidant effect of the compounds isolated from *A. debrana* roots and standard (DPPH and H_2_O_2_) assays.

Assay	Compounds/standard					
	**1**	**2**	**3**	**5**	**6**	AA
DPPH (IC_50_^*∗*^, *μ*g/mL)	>100	>100	19.38 ± 0.64	57.24 ± 1.07	32.81 ± 0.78	15.37 ± 0.44
H_2_O_2_ (IC_50_^*∗*^, *μ*g/mL)	>100	>100	28.52 ± 1.08	49.34 ± 1.33	27.31 ± 1.46	12.64 ± 0.92

Values are expressed as mean ± SD (*n* = 3). ^*∗*^IC_50_, 50% inhibitory concentration; DPPH, 2,2-diphenyl-1-picrylhydrazine; H_2_O_2_, hydrogen peroxide; **1**–**3**, **5**, **6,** isolated compounds; AA, ascorbic acid (positive control).

**Table 4:** Antibacterial activity of the compounds isolated from *A. debrana* roots.

Compounds	Concentration (mg/mL)	Percent inhibition of the bacterial growth (%)	
		Gram+	Gram−
		*Staphylococcus aureus*	*Escherichia coli*
Compound **1**	0.25	15.71 ± 0.08	NI
	0.5	32.97 ± 0.19	4.51 ± 0.95
	0.75	43.07 ± 0.33	6.25 ± 0.91
	1.0	54.27 ± 0.25	—

Compound **2**	0.25	13.82 ± 0.27	NI
	0.5	37.77 ± 0.09	3.42 ± 1.93
	0.75	55.93 ± 0.26	5.17 ± 1.52
	1.0	73.47 ± 0.05	—

Compound **3**	0.25	39.90 ± 0.15	NI
	0.5	57.49 ± 0.21	4.54 ± 0.91
	0.75	69.25 ± 0.18	7.05 ± 1.13
	1.0	91.20 ± 0.12	—

Compound **5**	0.25	39.67 ± 0.12	NI
	0.5	54.83 ± 0.17	3.06 ± 1.10
	0.75	66.09 ± 0.14	5.12 ± 0.72
	1.0	87.83 ± 0.05	—

Compound **6**	0.25	20.01 ± 0.08	NI
	0.5	35.57 ± 0.20	NI
	0.75	61.18 ± 0.08	NI
	1.0	78.02 ± 0.08	—

Gentamicin	0.75	88.90 ± 0.17	87.63 ± 0.27

Results are presented as mean ± SD (*n* = 3). NI, no inhibition; —, not tested.

**Table 5:** Diameter of inhibition zones of compounds **3** and **5**.

Compounds	Concentration (mg/mL)	DIZ (mm)	
		Gram+	Gram−
		*Staphylococcus aureus*	*Escherichia coli*
Compound **3**	0.5	6.87 ± 0.93	NI
	1.0	9.14 ± 0.93	2.91 ± 1.06

Compound **5**	0.5	6.55 ± 0.87	NI
	1.0	8.21 ± 1.24	NI

Gentamicin	1.0	24.74 ± 0.81	21.30 ± 0.69

Results are presented as mean ± SD (*n* = 3). DIZ, diameter of inhibition zone; NI, no inhibition.

## Data Availability

The images of the plant samples and acetone extract, and the NMR spectra used for the interpretation of compounds **3**, **5,** and **6** are included as supporting information files. The NMR spectra of the compounds **1**, **2,** and **4** and other data used to support the findings of this study are available from the corresponding author upon request.

## Supplementary Materials

Supplementary Materials In the supporting information files, the images of fresh, chopped and dried, and grinded powder roots of *A. debrana* is annexed as Figure S1, whereas the image of the acetone extract of the dried and powdered roots of *A. debrana* as Figure S2. Moreover, the ^1^H-, ^13^C-, DEPT-135, COSY, HSQC, HMBC, and mass spectra generated for the characterization of compound **3** as 5-allyl-3-methoxybenzene-1,2-diol are annexed as Figure S3, S4, S5, S6, S7, S8, and S9, respectively. Furthermore, the NMR spectra generated for identifying compound **5** as aloesaponarin I and compound **6** as laccaic acid D-methyl ester are annexed as Figure S10–S15 and Figure S16-S21, respectively. Figure S10: ^1^H-NMR spectrum of compound **5**. Figure S11: ^13^C-NMR spectrum of compound **5**. Figure S12: DEPT-135 NMR spectrum of compound **5**. Figure S13: COSY NMR spectrum of compound **5**. Figure S14: HSQC spectrum of compound **5**. Figure S15: HMBC spectrum of compound **5**. Figure S16: ^1^H-NMR spectrum of compound **6**. Figure S17: ^13^C-NMR spectrum of compound **6**. Figure S18: DEPT-135 NMR spectrum of compound **6**. Figure S19: COSY NMR spectrum of compound **6**. Figure S20: HSQC spectrum of compound **6**. Figure S21: HMBC spectrum of compound **6**.
