# Supplementary material for: Antibacterial and Antioxidant Compounds from the Root Extract of Aloe debrana
Source: Evid Based Complement Alternat Med. 2024 May 7;2024:6651648. doi: 10.1155/2024/6651648 (PMC11093683; doi:10.1155/2024/6651648)
Supplement: Supplementary Materials — In the supporting information files, the images of fresh, chopped and dried, and grinded powder roots of A. debrana is annexed as Figure S1, whereas the image of the acetone extract of the dried and powdered roots of A. debrana as Figure S2. Moreover, the 1H-, 13C-, DEPT-135, COSY, HSQC, HMBC, and mass spectra generated for the characterization of compound 3 as 5-allyl-3-methoxybenzene-1,2-diol are annexed as Figure S3, S4, S5, S6, S7, S8, and S9, respectively. Furthermore, the NMR spectra generated for identifying compound 5 as aloesaponarin I and compound 6 as laccaic acid D-methyl ester are annexed as Figure S10–S15 and Figure S16-S21, respectively. Figure S10: 1H-NMR spectrum of compound 5. Figure S11: 13C-NMR spectrum of compound 5. Figure S12: DEPT-135 NMR spectrum of compound 5. Figure S13: COSY NMR spectrum of compound 5. Figure S14: HSQC spectrum of compound 5. Figure S15: HMBC spectrum of compound 5. Figure S16: 1H-NMR spectrum of compound 6. Figure S17: 13C-NMR spectrum of compound 6. Figure S18: DEPT-135 NMR spectrum of compound 6. Figure S19: COSY NMR spectrum of compound 6. Figure S20: HSQC spectrum of compound 6. Figure S21: HMBC spectrum of compound 6. [file 6651648.f1.pdf]

Supporting Information

Antibacterial and antioxidant compounds from the roots extract of *Aloe debrana*

Tokuma Getahun,[1,*] Joydeep Das,[2] Parames C. Sil,[3] and Neeraj Gupta[4]

[1]*Department of Chemistry, Asella College of Teachers Education, Asella, Oromia, Ethiopia*

[2]*Department of Chemistry, Physical Sciences, Mizoram University, Aizawl, Mizoram 796004, India*

[3]*Division of Molecular Medicine, Bose Institute, P-1/12, CIT Scheme VII M, Kolkata 700054, India*

[4]*Department of Chemistry and Chemical Sciences, Central University of Himachal Pradesh, Dharamshala, Kangra 176215, HP, India*

[*]Corresponding author.

List of Supporting Information

Figure S1. The fresh, chopped and dried, and grinded powder roots of *A. debrana* 2

Figure S2. Acetone extract of the dried and powdered roots of *A. debrana* 2

Figure S3. ^{1}H−NMR (500 MHz, $CDCl_3$) spectrum of compound **3** 3

Figure S4. ^{13}C−NMR (125 MHz, $CDCl_3$) spectrum of compound **3** 3

Figure S5. DEPT (125 MHz, $CDCl_3$) spectrum of compound **3** 4

Figure S6. ^{1}H−^{1}H COSY (500 MHz, $CDCl_3$) spectrum of compound **3**.....................................4

Figure S7. HSQC ($CDCl_3$) spectrum of compound **3** (^{1}H: 500 MHz, ^{13}C: 125 MHz) 5

Figure S8. HMBC ($CDCl_3$) spectrum of compound **3** (^{1}H: 500 MHz, ^{13}C: 125 MHz) 5

Figure S9. Mass spectrum of compound **3** 6

Figure S10. ^{1}H−NMR (500 MHz, DMSO-d_6) spectrum of compound **5** 6

Figure S11. ^{13}C−NMR (125 MHz, DMSO-d_6) spectrum of compound **5** 7

Figure S12. DEPT (125 MHz, DMSO-d_6) spectrum of compound **5** 7

Figure S13. ^{1}H−^{1}H COSY (500 MHz, DMSO-d_6) spectrum of compound **5**..............................8

Figure S14. HSQC (DMSO-d_6) spectrum of compound **5** (^{1}H: 500 MHz, ^{13}C: 125 MHz)..........8

Figure S15. HMBC (DMSO-d_6) spectrum of compound **5** (^{1}H: 500 MHz, ^{13}C: 125 MHz) 9
Figure S16. ^{1}H−NMR (500 MHz, DMSO-d_6) spectrum of compound **6** 9
Figure S17. ^{13}C−NMR (125 MHz, DMSO-d_6) spectrum of compound **6** 10
Figure S18. DEPT (125 MHz, DMSO-d_6) spectrum of compound **6** 10
Figure S19. ^{1}H−^{1}H COSY (500 MHz, DMSO-d_6) spectrum of compound **6**. 11
Figure S20. HSQC (DMSO-d_6) spectrum of compound **6** (^{1}H: 500 MHz, ^{13}C: 125 MHz) 11
Figure S21. HMBC (DMSO-d_6) spectrum of compound **6** (^{1}H: 500 MHz, ^{13}C: 125 MHz) 12

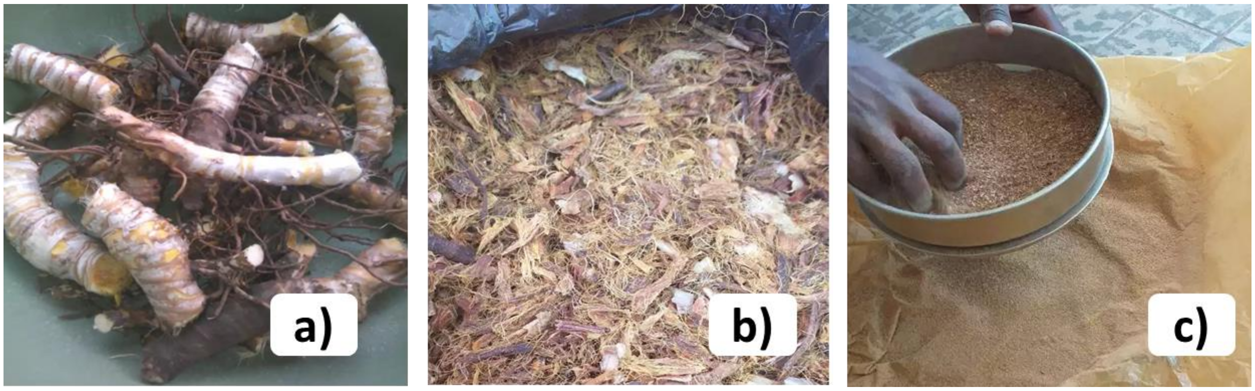

Figure S1. The fresh (a), chopped and dried (b) and grinded powder (c) roots of *A. debrana*

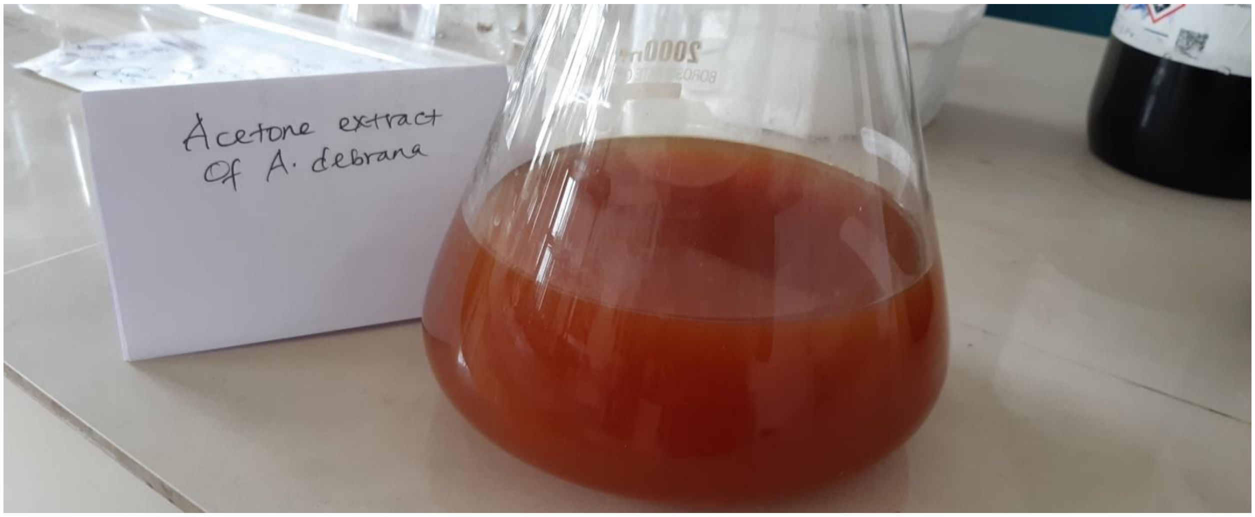

Figure S2. Acetone extract of the dried and powdered roots of *A. debrana*

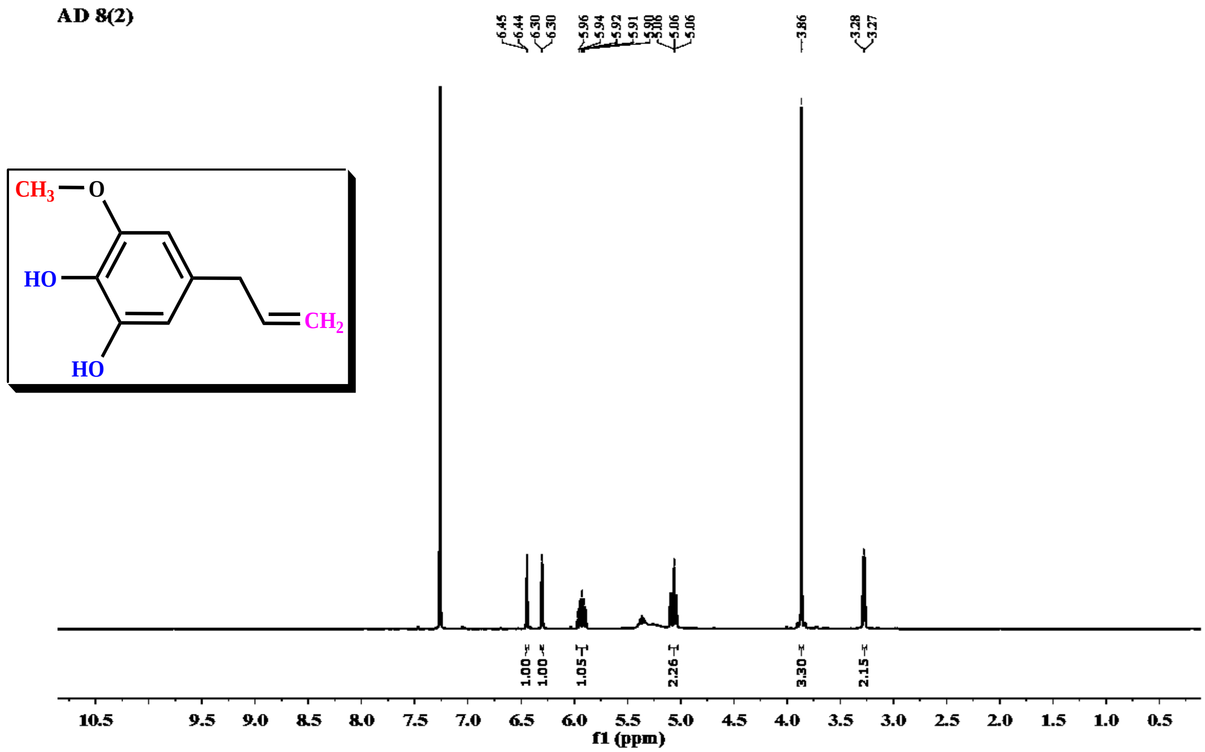

Figure S3. ^{1}H−NMR (500 MHz, $CDCl_3$) spectrum of compound **3**

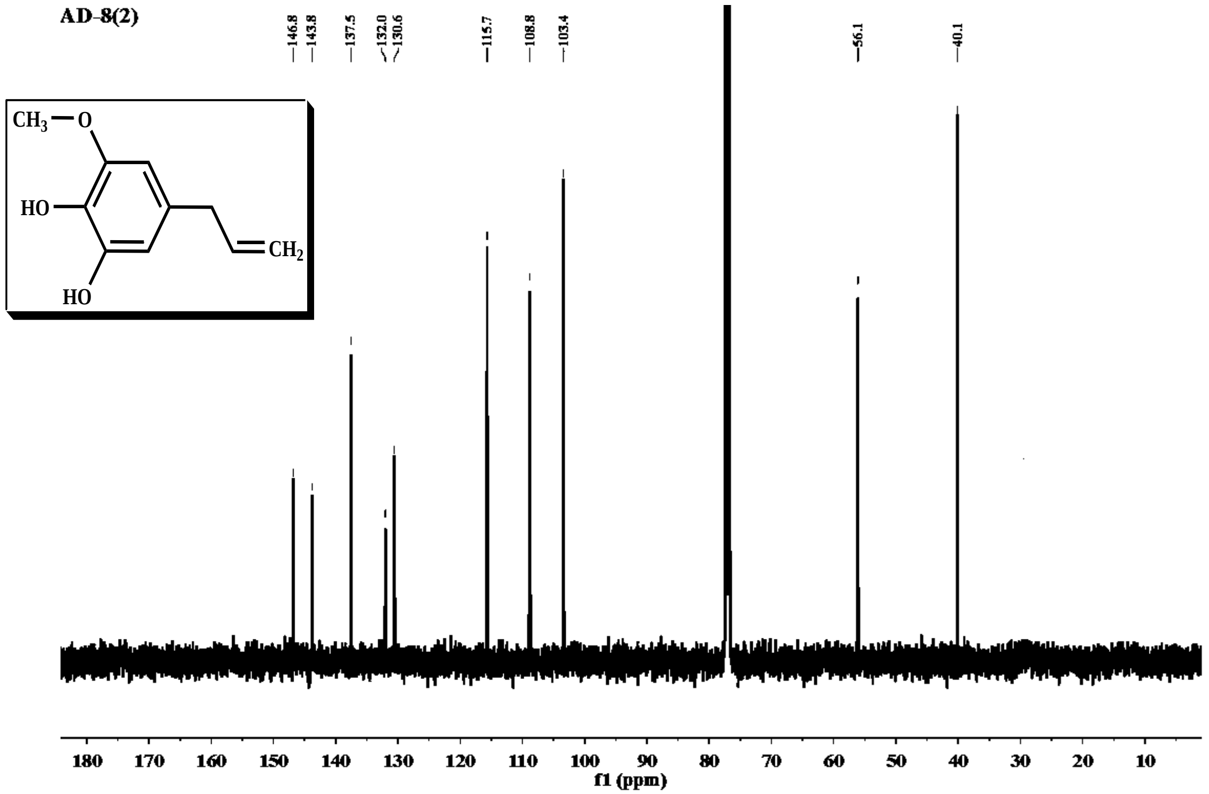

Figure S4. ^{13}C−NMR (125 MHz, $CDCl_3$) spectrum of compound **3**

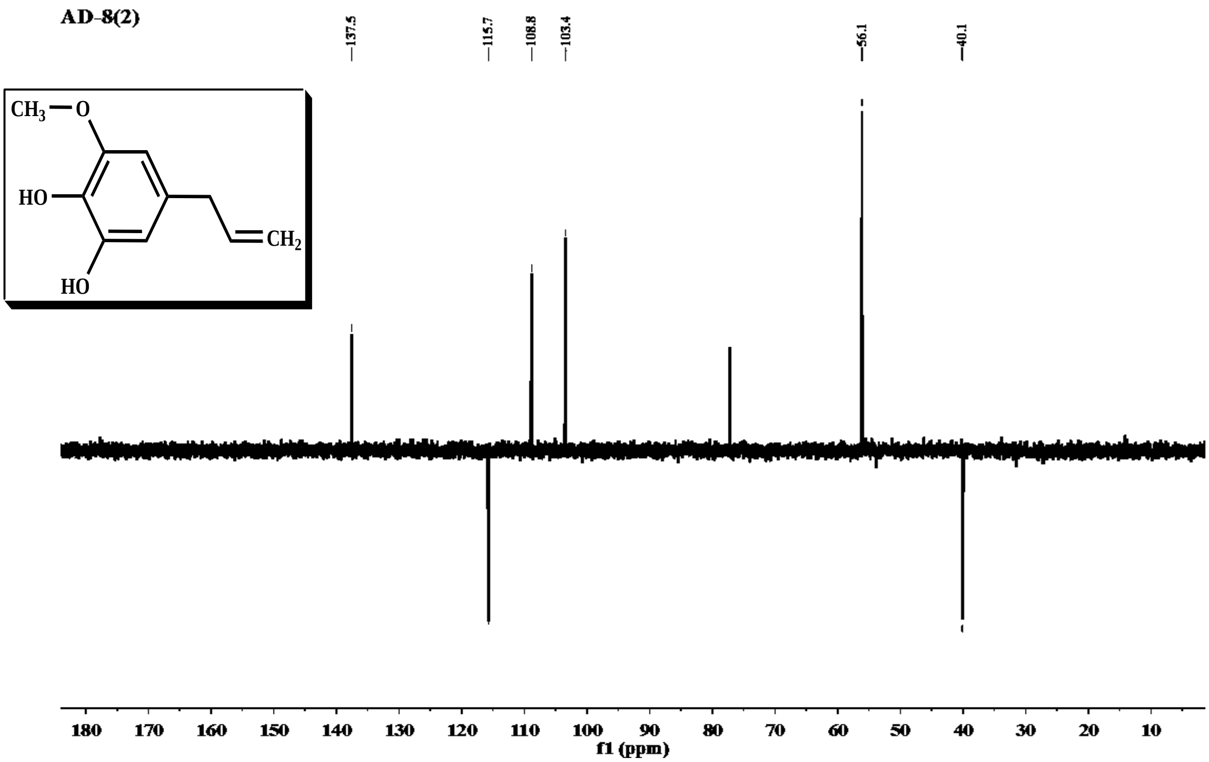

Figure S5. DEPT (125 MHz, $CDCl_3$) spectrum of compound **3**

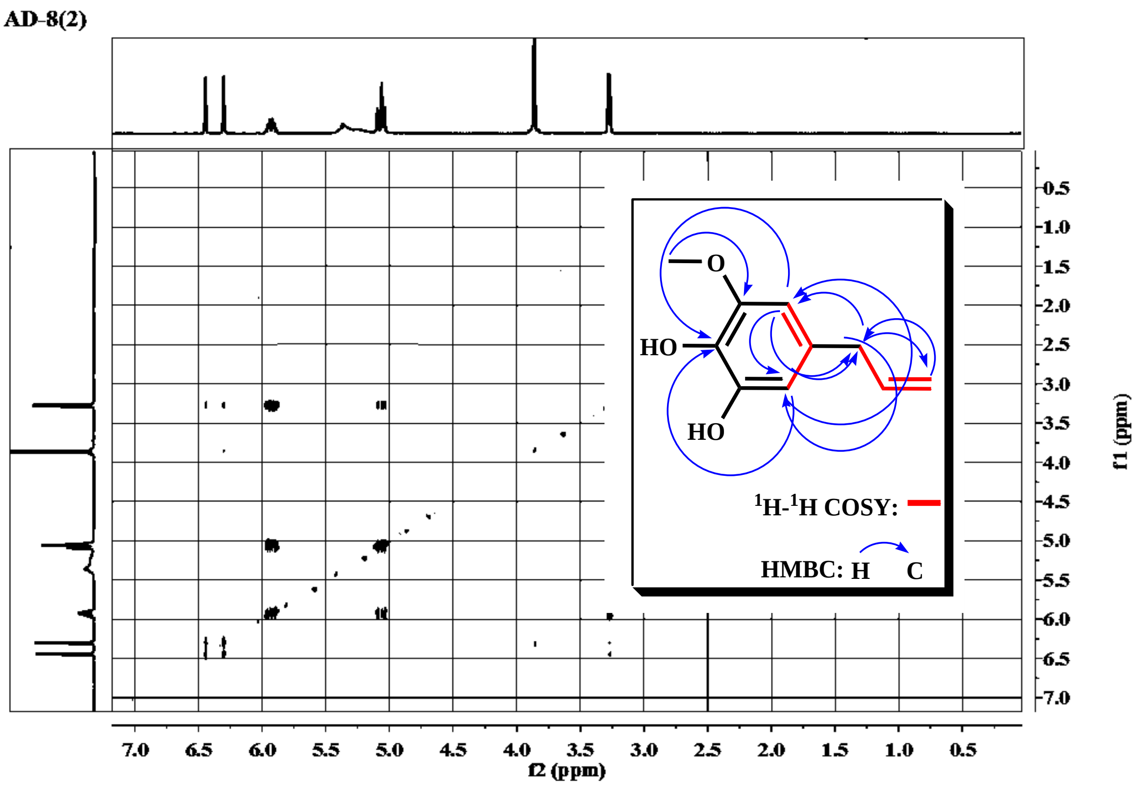

Figure S6. $^1H-^1H$ COSY (500 MHz, $CDCl_3$) spectrum of compound **3**

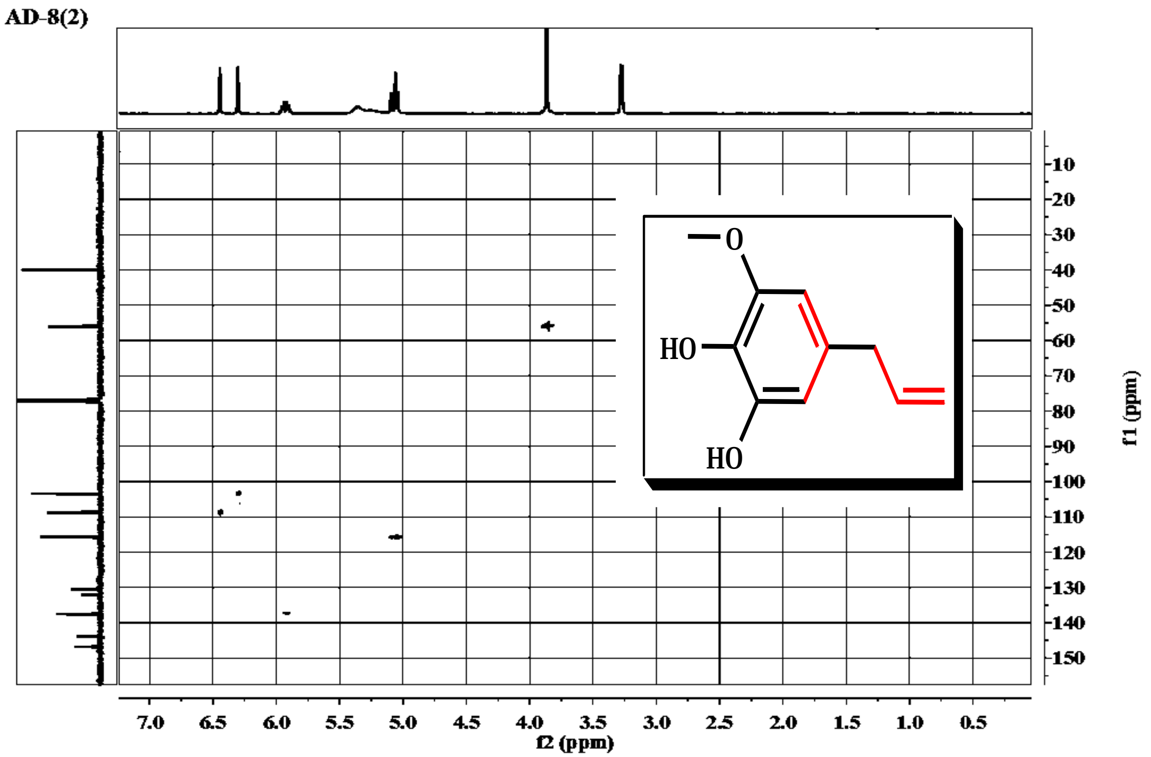

Figure S7. HSQC (CDCl3) spectrum of compound **3** (^{1}H: 500 MHz, ^{13}C: 125 MHz)

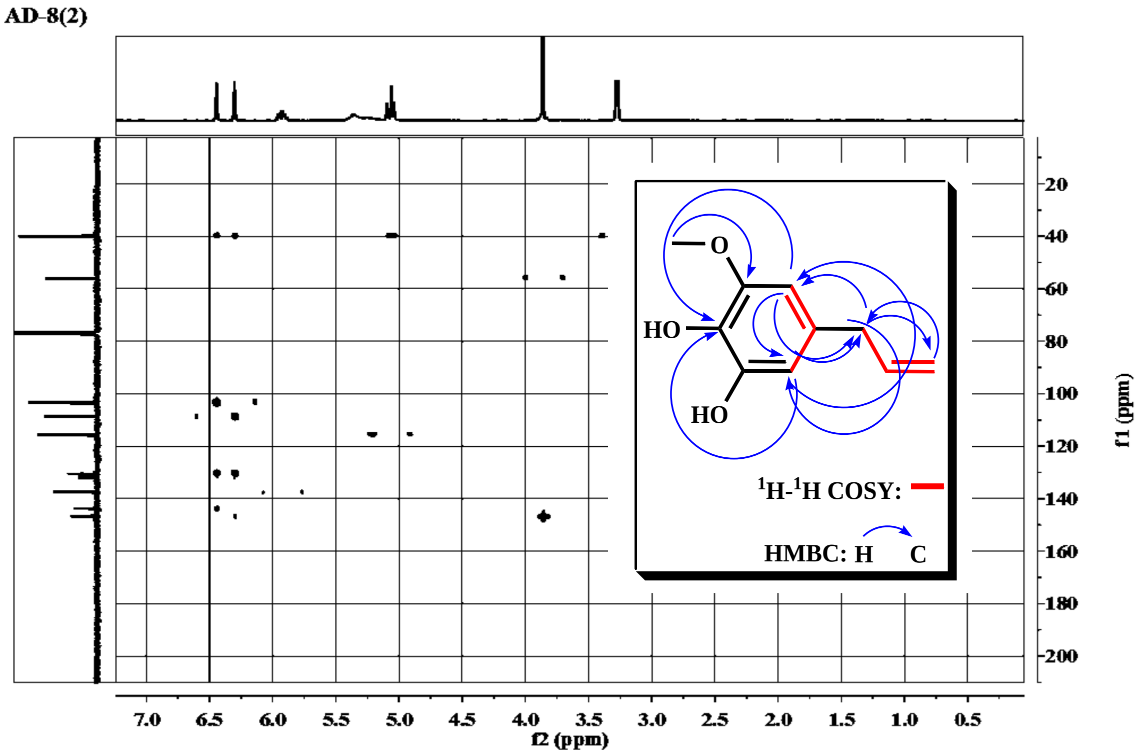

Figure S8. HMBC (CDCl3) spectrum of compound 3 (^{1}H: 500 MHz, ^{13}C: 125 MHz)

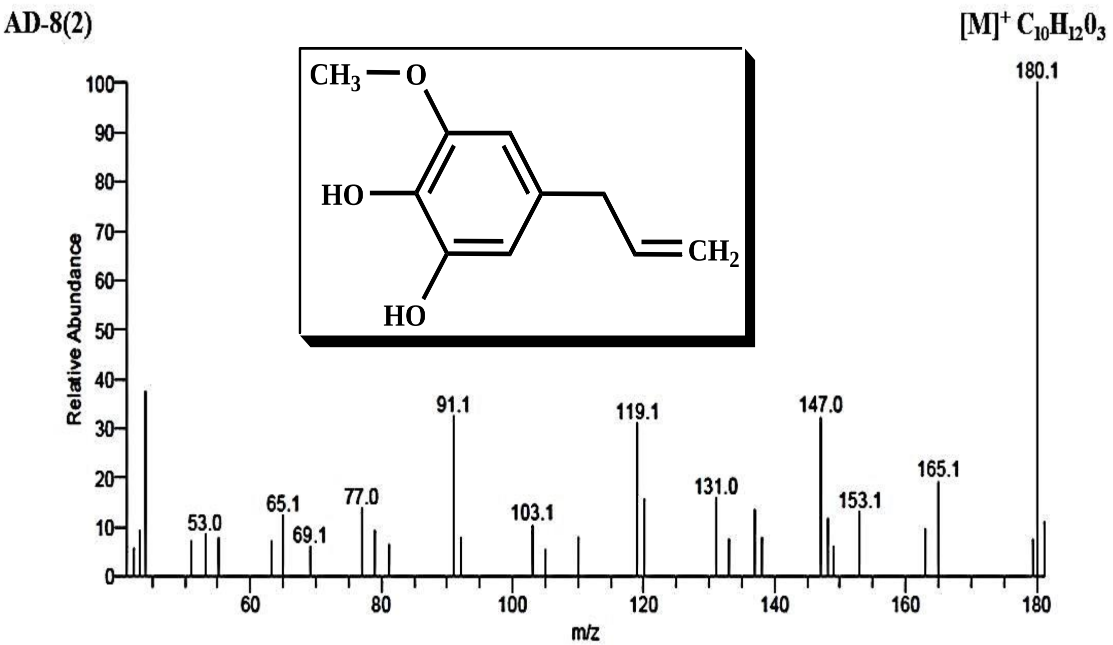

Figure S9. Mass spectrum of compound **3**

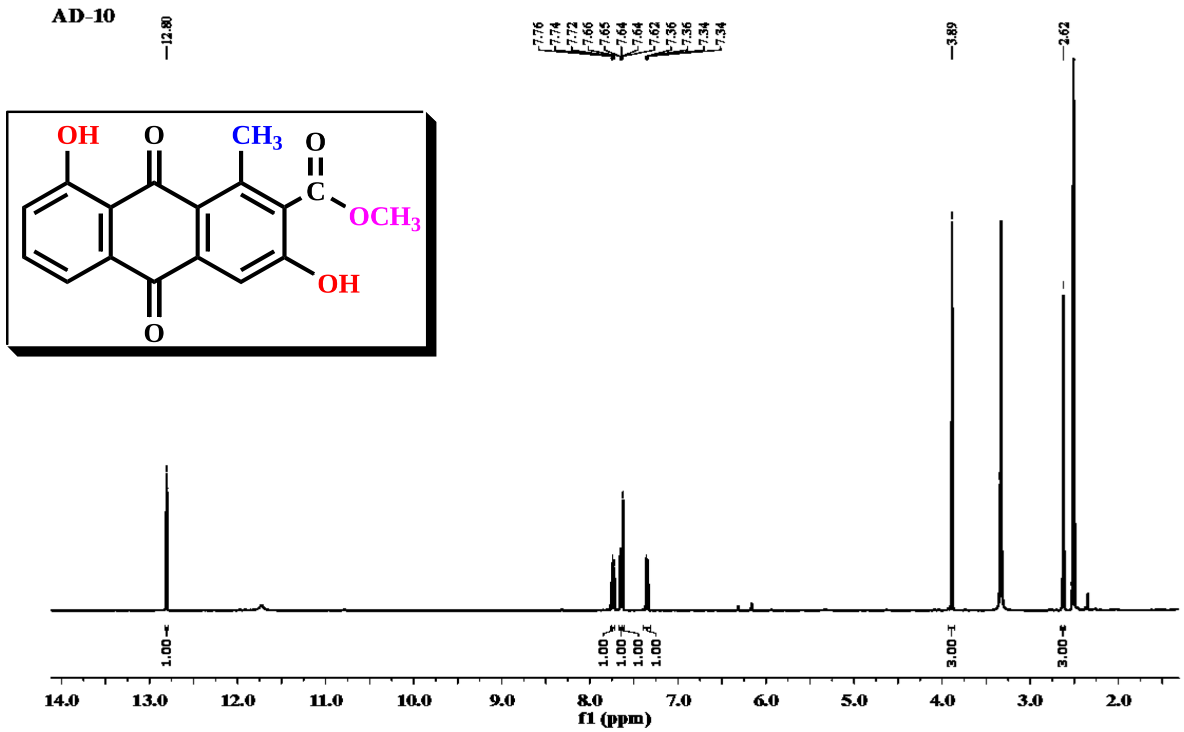

Figure S10. ^{1}H−NMR (500 MHz, DMSO-d_6) spectrum of compound **5**

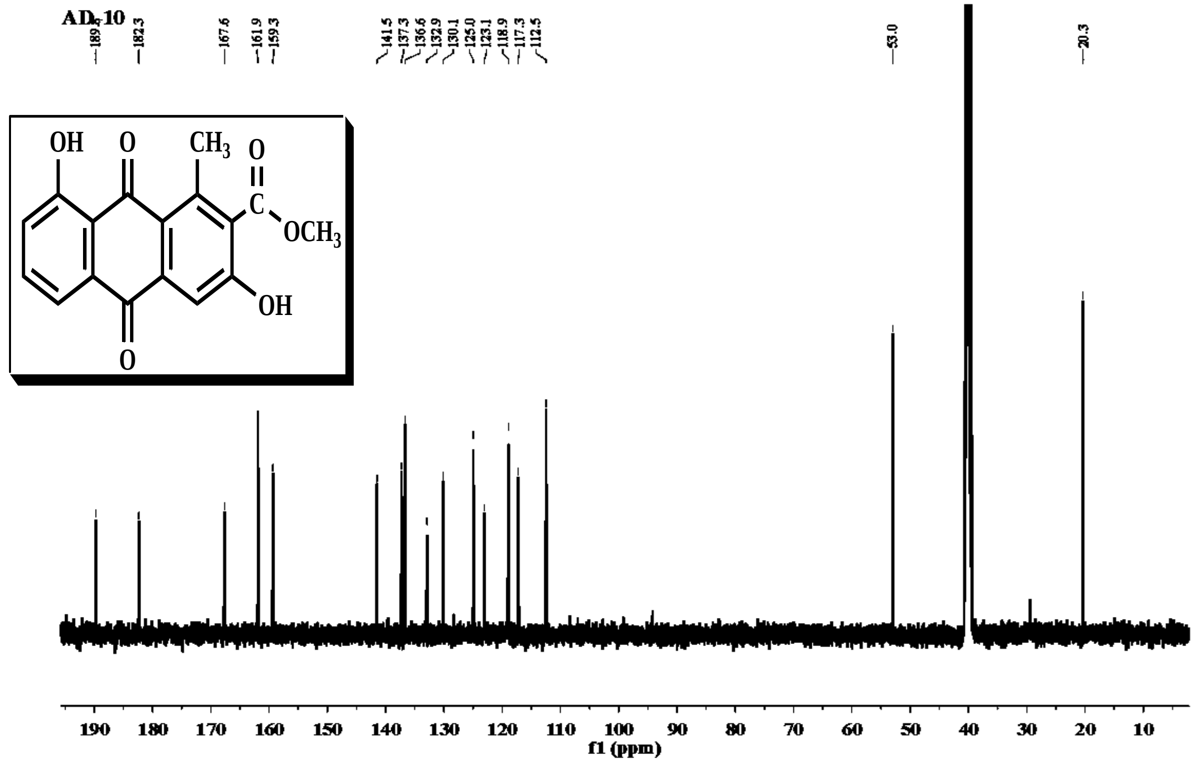

Figure S11. ^{13}C−NMR (125 MHz, DMSO-d_6) spectrum of compound **5**

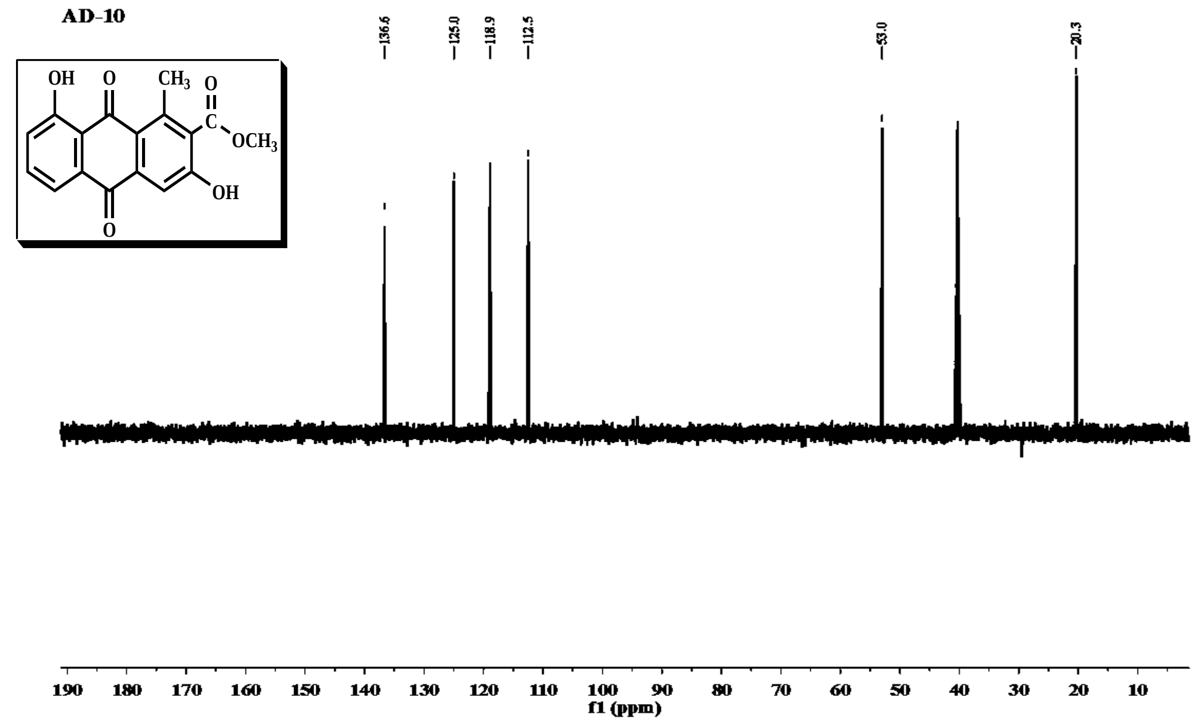

Figure S12. DEPT (125 MHz, DMSO-d_6) spectrum of compound **5**

AD-10

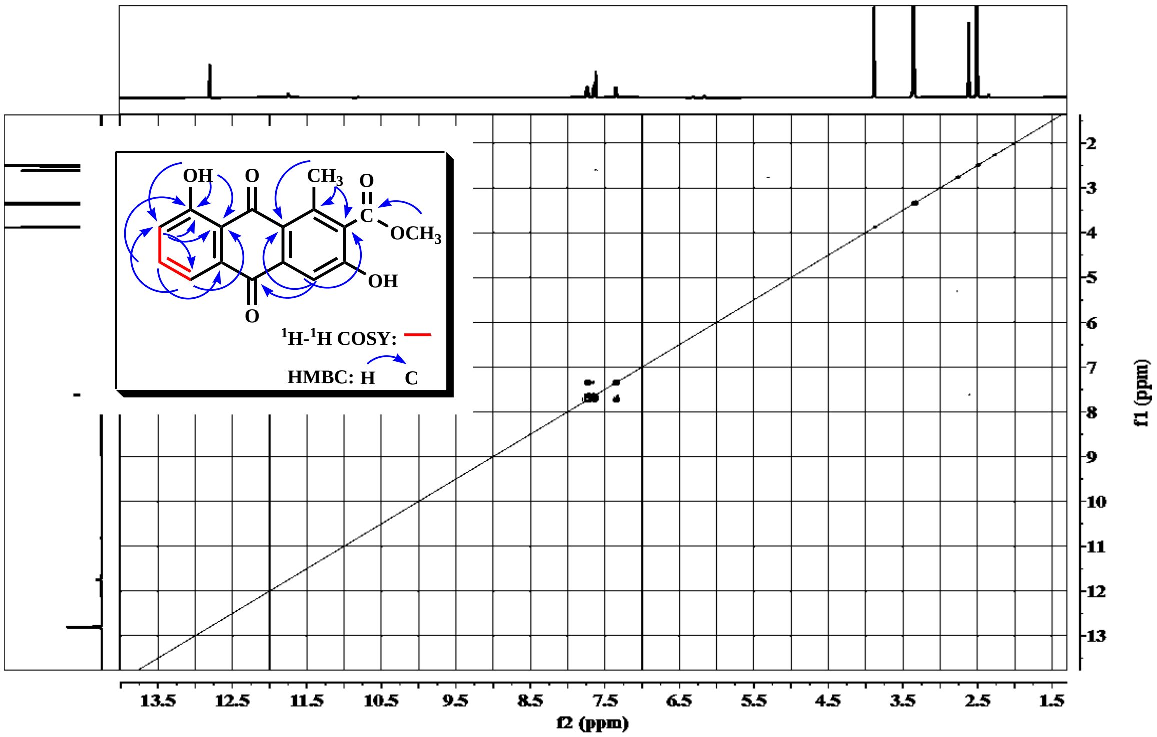

Figure S13. $^{1}H-^{1}H$ COSY (500 MHz, DMSO-d_6) spectrum of compound **5**

AD-10

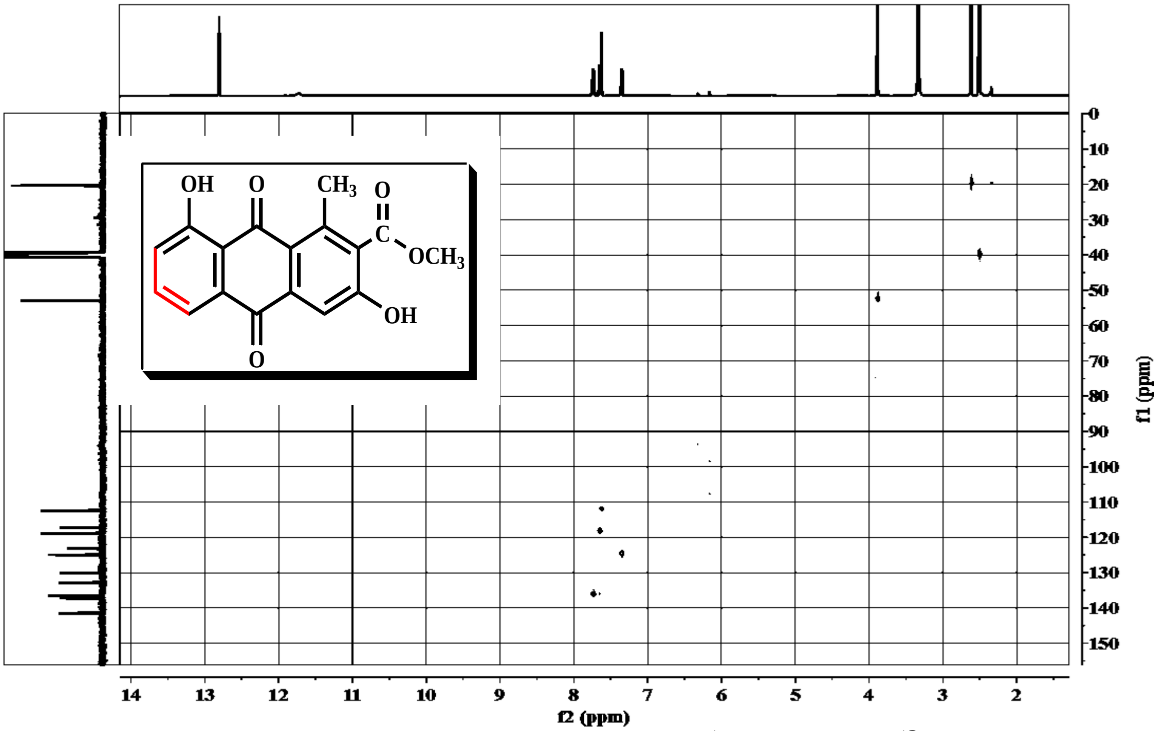

Figure S14. HSQC (DMSO-d_6) spectrum of compound **5** (^{1}H: 500 MHz, ^{13}C: 125 MHz)

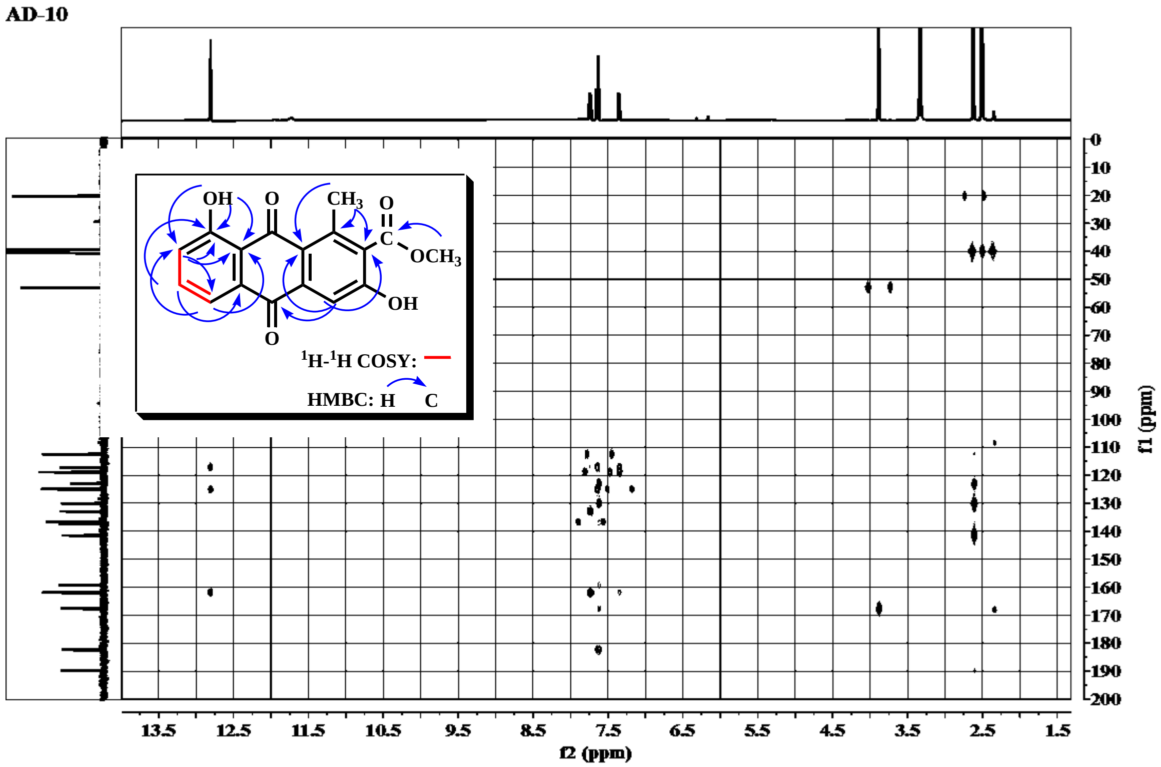

Figure S15. HMBC (DMSO-d_6) spectrum of compound **5** (^{1}H: 500 MHz, ^{13}C: 125 MHz)

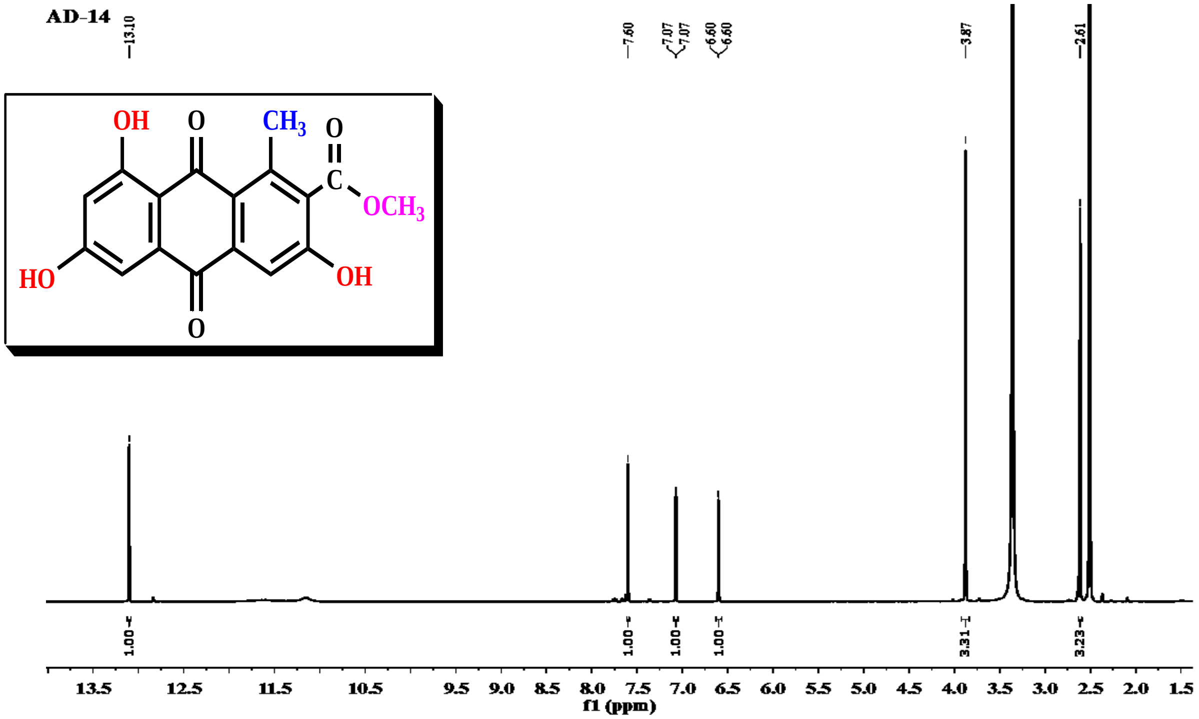

Figure S16. ^{1}H−NMR (500 MHz, DMSO-d_6) spectrum of compound **6**

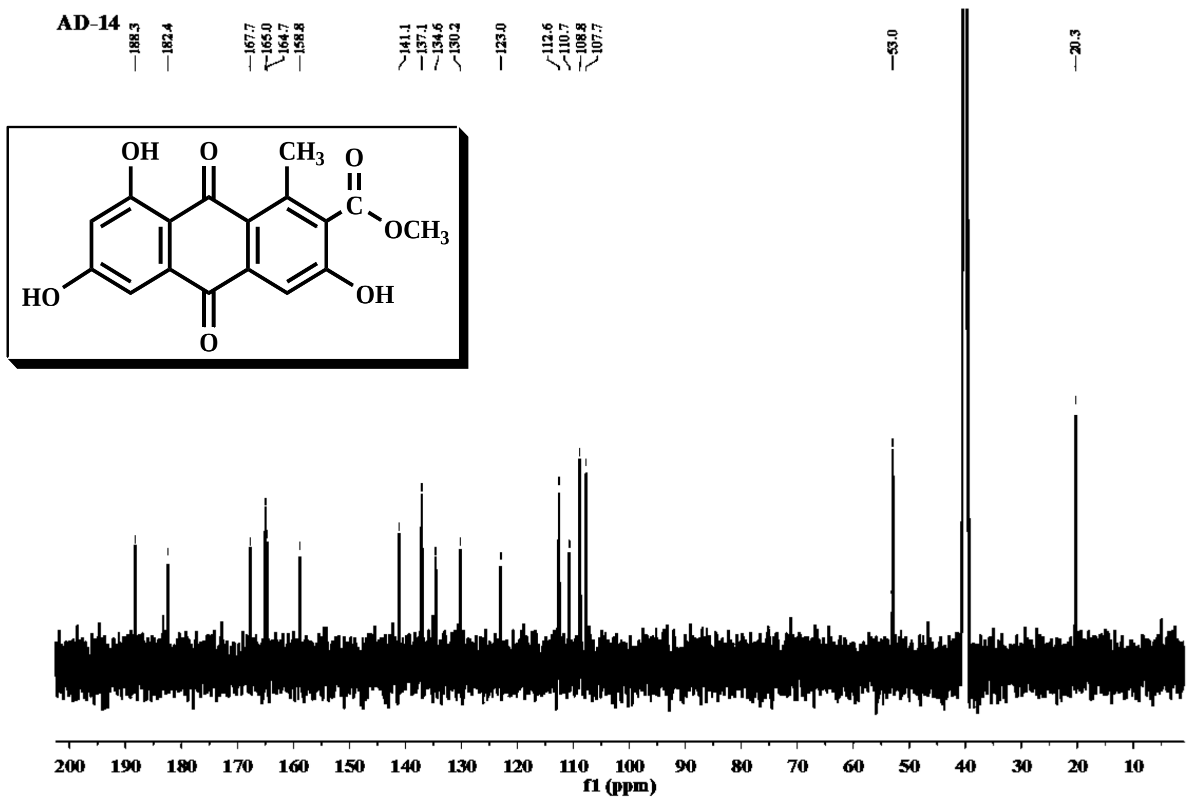

Figure S17. ^{13}C−NMR (125 MHz, DMSO-d_6) spectrum of compound **6**

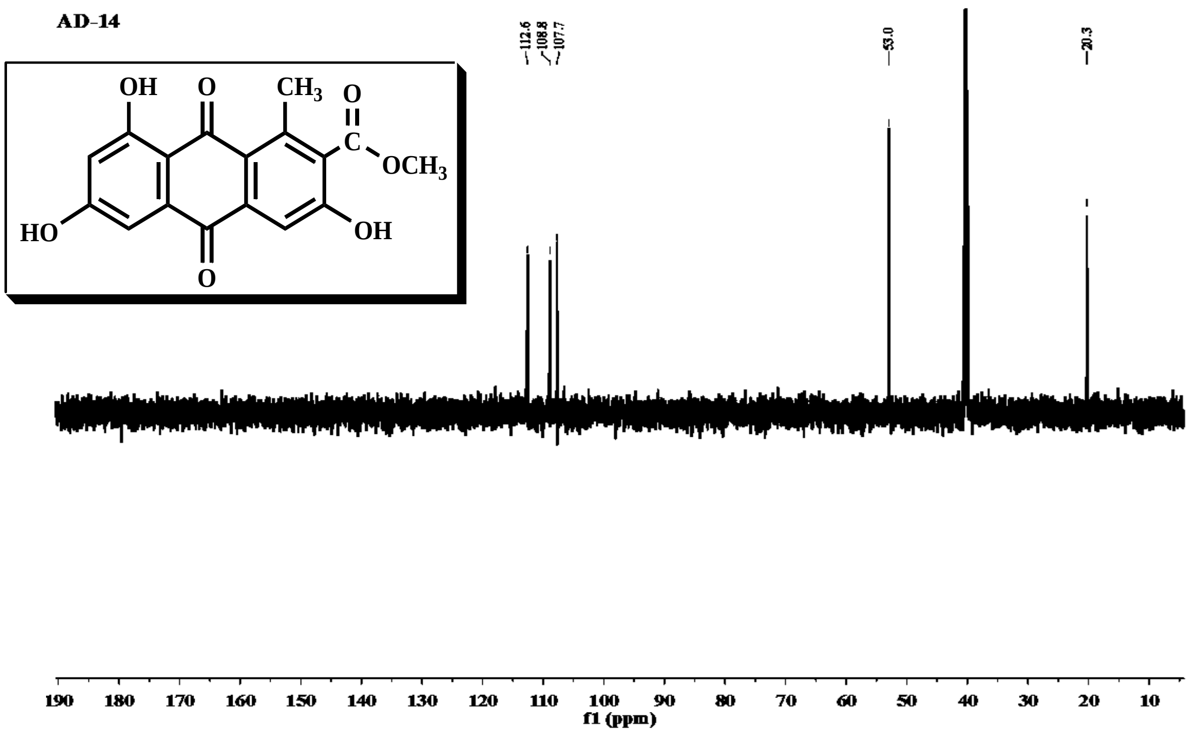

Figure S18. DEPT (125 MHz, DMSO-d_6) spectrum of compound **6**

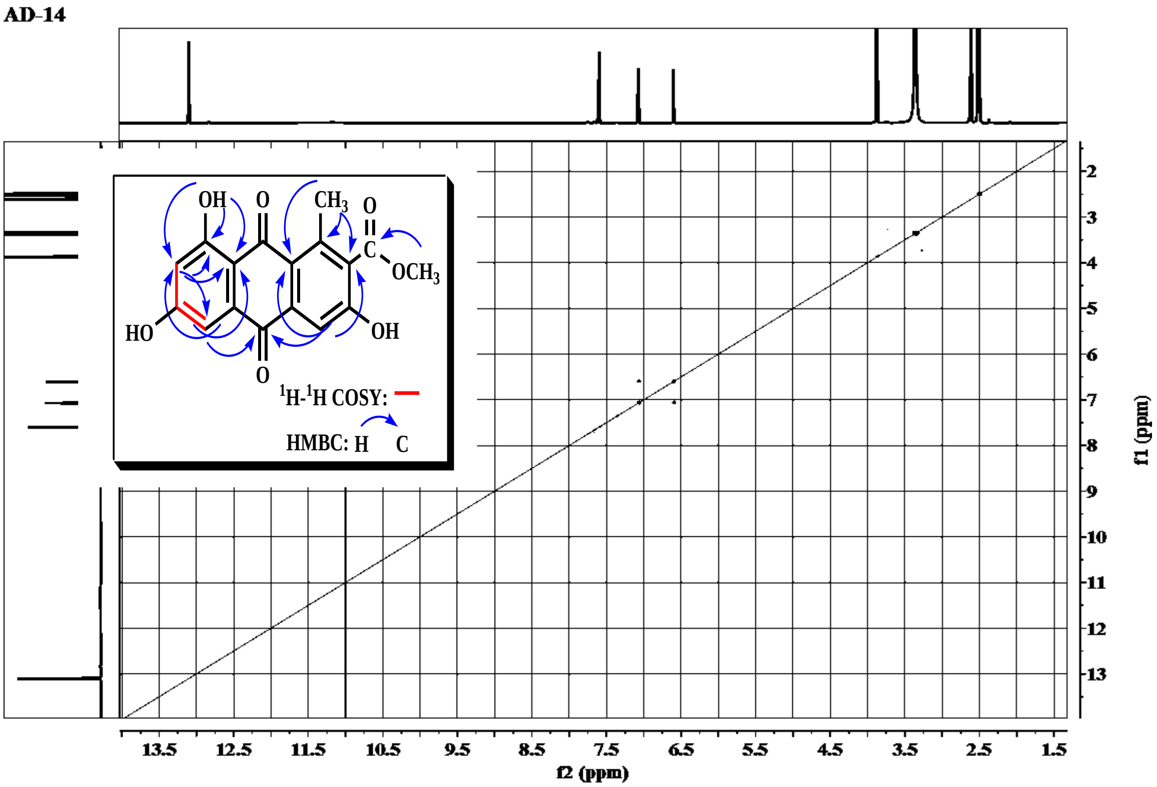

Figure S19. ^1H–^1H COSY (500 MHz, DMSO-d_6) spectrum of compound **6**

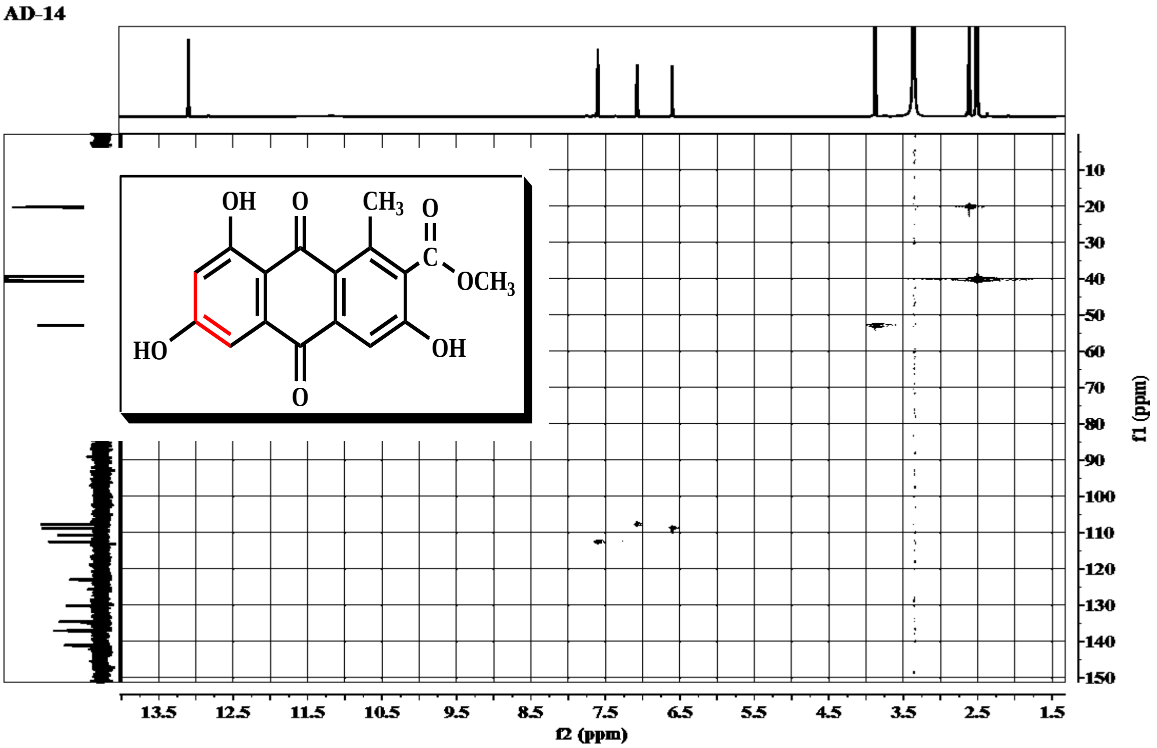

Figure S20. HSQC (DMSO-d_6) spectrum of compound **6** (^1H: 500 MHz, ^{13}C: 125 MHz)

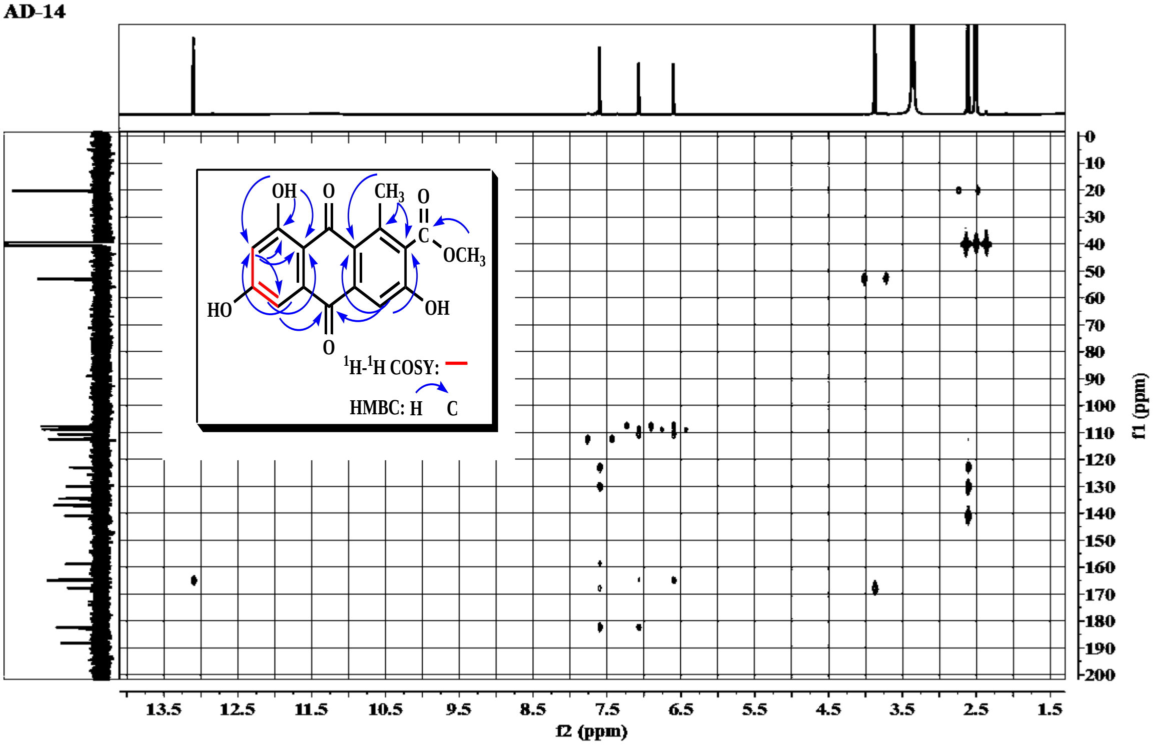

Figure S21. HMBC (DMSO-d_6) spectrum of compound **6** (^{1}H: 500 MHz, ^{13}C: 125 MHz)
